# Impact of screening on late-stage breast cancer in the Netherlands: a population-based cohort study (2007-2016)

**DOI:** 10.1016/j.breast.2026.104745

**Published:** 2026-03-02

**Authors:** Yixuan Lin, Grigory Sidorenkov, Jing Wang, Marcel J.W. Greuter, Nehmat Houssami, Monique D. Dorrius, Sabine Siesling, Linda de Munck, Geertruida H. de Bock

**Affiliations:** a Department of Epidemiology, University of Groningen, University Medical Center Groningen, Groningen, the Netherlands; b Department of Epidemiology and Health Statistics, School of Public Health, Hangzhou Medical College, Hangzhou, China; c Department of Radiology, University of Groningen, University Medical Center Groningen, Groningen, the Netherlands; d The Daffodil Centre, The University of Sydney, a Partnership with Cancer Council NSW, Sydney, NSW, Australia; e Department of Research and Development, Netherlands Comprehensive Cancer Organisation (IKNL), Utrecht, the Netherlands; f Department of Health Technology and Services Research, Technical Medical Centre, University of Twente, Enschede, the Netherlands

**Keywords:** Breast neoplasms, Incidence, Cancer screening

## Abstract

**Background:**

Breast cancer (BC) screening can detect BC early to reduce mortality. This study evaluated the impact of BC screening on the incidence of late-stage BC in a population-based setting in the Netherlands.

**Methods:**

All Dutch women aged 50–74 years diagnosed with invasive BC or ductal carcinoma in situ (DCIS) between 2007 and 2016 (n = 108,253) were included from the Netherlands Cancer Registry. BC was classified as screen-related if diagnosed within 24 months the last screening attendance, and as screen-detected if diagnosed within 12 months after positive screening result. Late-stage BC was defined in two ways: advanced BC, including TNM stages T3, T4, or N2, N3, or M1, and metastatic BC, defined as M1 disease. Multivariable logistic regression adjusted for age and socioeconomical status was used to assess associations between screen-related and screen-detected BC and late-stage BC. Analyses were done overall and stratified by HR/HER2-defined subtypes.

**Findings:**

BC incidence increased between 2007 and 2013 and decreased slightly thereafter. Advanced BC incidence decreased between 2007 and 2016, while metastatic BC rates remained stable. Non-screen-related BCs were significantly more likely to be present as late disease compared with screen-related (advanced BC: aOR = 3.24, 95%CI = 3.12–3.37; metastatic BC: aOR = 6.40, 95%CI = 5.98-6.85). Similarly, non-screen-detected BCs had substantial higher odds of being late than screen-detected BCs (advanced BC: aOR = 5.54, 95%CI = 5.31–5.78; metastatic BC: aOR = 12.66, 95% CI = 11.41-14.05) than screen-detected BCs. These associations were observed across all HR/HER2-defined subtypes.

**Interpretation:**

Population-based screening is strongly associated with earlier-stage breast cancer at diagnosis, consistently across all immunohistochemistry subtypes.

## Highlights

• This nationwide study used individual-level data to assess screening effectiveness.
• Non-screen-related breast cancers are more likely to be late-stage breast cancers.
• Screen-related breast cancers have an earlier stage in all immunochemistry subtypes.

## 1 Introduction

Breast cancer (BC) is the most prevalent cancer in women, with an annual incidence of 46.3 per 100,000 women in 2018 [1]. The Netherlands has the second highest BC incidence worldwide, with 153.1 cases of ductal carcinoma in situ (DCIS) or invasive BC per 100,000 women in 2022 [2]. Therefore, as with other countries, the Netherlands implemented a national BC Screening Program (BCSP) to detect and treat disease early [3]. In Europe, regular BC screening has been associated with 4% to 31% reductions in mortality [4]. However, debates persist regarding how effectively BC screening reduces mortality, with some attributing improved survival more to advancements in BC treatment [5].

Although measuring BC mortality is useful for assessing outcomes, it is also possible to use the incidence of late-stage BC at initial diagnosis as a surrogate for screening effectiveness [6]. Some studies report 17% to 25% reductions in late-stage BC [[7], [8], [9], [10]], while others show inconclusive results [9,10], likely due to differences in study design, definitions of late-stage BC and analysis [11]. Moreover, BC is a heterogeneous disease, and few studies have examined the association between mode of detection and stage at diagnosis; and potential effect on immunohistochemistry-based subtype has been insufficiently studied. Furthermore, factors that may have underestimated screening benefits include short observation periods, use of aggregated data, imprecise screening status, and low participation. For example, differences in the definition of late-stage BC have influenced findings; although tumour stage is determined by TNM classification, many studies have defined early and late stages based on T-stage alone, without considering the number of lymph nodes or metastases [[11], [12], [13], [14]]. Few studies have also linked individual screening status to BC stage at diagnosis [13,[15], [16], [17]]. Many studies compared the incidence before and after screening program implementation, ignoring changes in background risk over the decades. They also assumed that all women participated in screening, even though participation rates were not high. In a previous study, a steady rise was observed in BC incidence in lower stage coupled with a stable incidence of late-stage BC between 2006 and 2011 in the Netherlands; however, evaluating the incidence over a short timeframe may not have adequately captured the full impact of the screening [17,18].

The aim of this study was to evaluate the impact of the Dutch national BCSP on the incidence and subtypes of late-stage BC. Data from 2007 to 2016 were analyzed for women aged 49–74 years diagnosed with early or late-stage BC (including DCIS and invasive BC). We hypothesized that BCs not related to screening are more likely to be at late-stage compared to BCs related to screening.

## 2 Methods

### 2.1 Context

The Dutch BCSP began in the mid-1970s and became nationwide by 1997, targeting women aged 50–69 years with biennial invitations. In 2001, screening was extended to women up to age 74 years [19]. Women turning 50 years old in a calendar year are invited to the BCSP, which is overseen by the National Institute for Public Health and the Environment (RIVM). BCSP attendance was 70.3% in 2023 [20].

#### 2.1.1 Study population and data collection

The Netherlands Cancer Registry (NCR) gathers data on patient, tumour, and treatment characteristics for tumours diagnosed in the Netherlands since 1989 [19]. Individual NCR data were linked to the BCSP database to determine the screening status. The following data from 2007 to 2016 for women aged 49–74 years diagnosed with DCIS or invasive BC were analyzed: age; socioeconomic status (SES); year of diagnosis; and tumour characteristics (clinical and pathological stage, metastasis, tumour size, and lymph node involvement, estrogen receptor (ER), progesterone receptor (PR), and human epidermal growth factor receptor 2 (HER2). Data on screening year, invitation and participation, interval in days between screening and diagnosis were available from the BCSP database. Population data were sourced from the CBS [21]. Analysis was limited to the last round of screening before the diagnosis date.

#### 2.1.2 Definitions

In the analysis screen-related as well as screen-detected were considered. Screen-related BC was defined as breast cancer diagnosed within 24 months after a woman's last known attendance at the BCSP, regardless of the screening result. This category included screen-detected cancers following a positive screening result and interval cancers diagnosed between regular screenings after an initial negative result. Because interval cancers are more likely to be detected at an earlier stage than cancers in irregular or never attenders, an association with screening was assumed [22]. Any remaining cases were classified as non-screen-related BC. A screen-detected BC was defined as BC diagnosed within 12 months after a woman's last known attendance at the BCSP with a positive screening result. All remaining BCs were classified as non-screen detected.

SES was measured using a neighbourhood-level postal code score provided by Central Bureau of Statistics (CBS), based on income, education, and employment, and categorized as low [[1], [2], [3]], medium [[4], [5], [6], [7]], or high [[8], [9], [10]] [23].

Late-stage BC was defined using two alternative definitions: advanced BC and metastatic BC. First, advanced BC was defined as T3–T4 disease (≥50 mm or involving the chest wall/skin), N2–N3 nodal involvement, or M1 (distant metastasis), based on an expert consensus publication [24]. All other tumours, including DCIS and invasive BC not meeting these criteria, were classified as early BC. Second, metastatic BC was defined more restrictively as M1 (distant metastasis) disease only. All other tumours, including DCIS and non-metastatic invasive BC, were classified as non-metastatic BC (M0) [25]. Tumours were classified according to the TNM system in use at the time of diagnosis (6th edition for diagnoses in 2007–2009 [26] and 7th edition for diagnoses from 2010 to 2016 [27]), and the thresholds for T3–T4, N2–N3, and M1 relevant to this study were consistent across these editions of the American Joint Committee on Cancer/Union for International Cancer Control TNM classification. For women with multiple tumours, the first diagnosed tumour or the highest stage (if diagnosed simultaneously) was used. Clinical tumour stage was used for women receiving neoadjuvant therapy and pathological tumour stage was used in all other cases.

BC immunohistochemistry subtypes were defined based on hormone receptor (HR) and Human Epidermal Growth Factor Receptor 2 (HER2) status. ER or PR status was considered positive if nuclear immunohistochemical staining was observed in more than 10% of tumour cells [28]. HR positivity was defined as positivity for either ER or progesterone receptor (PR), whereas HR negativity was defined as both ER and PR to be negative. Based on HR and HER2 status, tumours were classified into four subtypes: HR+/HER2+, HR+/HER2−, HR−/HER2+, and HR−/HER2−. In DCIS, HER2 status is generally not assessed and ER/PR testing may be optional in routine clinical practice [29], resulting in absent subtype data. Subtype information was also occasionally missing for invasive cancers.

### 2.2 Statistical analysis

The incidence of BC was reported as age-adjusted revised European standardized incidence rates (RESR) [30] with 95% confidence intervals (CIs), calculated by dividing the number of newly diagnosed cases per year by the number of women aged 50–74 years in the Netherlands in the same year, multiplied by 100,000. Population was averaged annually. Univariate logistic regression models evaluated the association of non-screen-related as well as non-screen-detected diagnosis with advanced BC and metastatic BC. The multivariable logistic models were adjusted for age and SES. In this way adjusted ORs (aORs) were estimated. The same multivariable logistic regression models were subsequently applied in stratified analyses by immunohistochemistry subtypes. This analysis was restricted to cases with recorded subtype. Results were reported as odds ratios (ORs) with 95% CIs. For interpretability, the reference category in the logistic regression analyses was set to screen-related/screen-detected BC, so that ORs reflect comparisons with non-screen-related or non-screen-detected BC. Analyses were performed in STATA 16.0.

### 2.3 Ethical considerations

This retrospective study was conducted with pseudomised and aggregated data from the Netherlands Cancer Registry/Netherlands Comprehensive Cancer Organisation. The study was approved by the privacy committee of the NCR as well as the NABON-BOOG scientific evaluation committee (reference number K20.389). According to Dutch legislation, this study does not fall under the scope of the Medical Research Involving Human Subjects Act (WMO) and therefore did not require approval from a medical ethics review committee.

## 3 Results

### 3.1 Participants

**Fig. 1:**
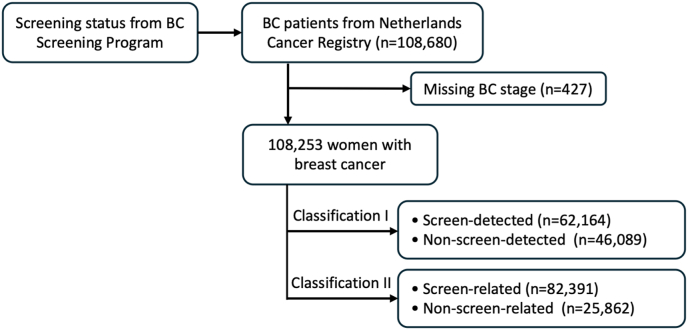
Flowchart of selection of study participants of this cohort study.

The cohort included 108,680 women aged 49–74 diagnosed with BC in the period 2007–2016; of these, 427 were excluded due to missing data on cancer stage (Fig. 1). Table 1 shows the characteristics of the women (n = 108,253) overall and stratified by screen-related and non-screen-related BC. BC diagnoses were more common in the 49–54-year age group compared with other age groups, and late-stage cancer was more common in the non-screened-related group (23.6% advanced BC and 9.6% metastatic BC) compared with the screen-related group (8.7% advanced BC and 1.7% metastatic BC). There were 62,164 women with screen-detected BC and 46,089 women with non-screen-detected BC. Table 2 compares the characteristics of these two groups. As with screen-related BC, BC was more common in women aged 49–54 and 65-69 years compared with other age groups in screen-detected BC, while it was only common in women aged 49–54 years in non-screen-detected BC, and late-stage cancer was more common in the non-screened-detected group (22.2% advanced BC and 7.5% metastatic BC) compared with the screen-detected group (4.9% advanced BC and 0.7% metastatic BC).

**Table 1:** Characteristics of women and their breast cancer by screen-related status.

	Total (n = 108,253)		Screen-related BC (n = 82,391)		Non-screen-related BC (n = 25,862)	
	N	%	N	%	N	%
**Age at diagnosis**						
49–54	27,794	25.7	18,628	22.6	9166	35.4
55–59	19,404	17.9	15,201	18.5	4203	16.3
60–64	22,125	20.4	17,514	21.3	4611	17.8
65–69	21,682	20.0	17,381	21.1	4301	16.6
70–74	17,248	15.9	13,667	16.6	3581	13.9
**Year of diagnosis**						
2007	9413	8.7	6999	8.5	2414	9.3
2008	9666	8.9	7195	8.7	2471	9.6
2009	9901	9.1	7304	8.9	2597	10.0
2010	10,293	9.5	7886	9.6	2407	9.3
2011	10,801	10.0	8310	10.1	2491	9.6
2012	11,315	10.5	8753	10.6	2562	9.9
2013	11,677	10.8	9010	10.9	2667	10.3
2014	11,640	10.8	8863	10.8	2777	10.7
2015	11,712	10.8	8983	10.9	2729	10.5
2016	11,835	10.9	9088	11.2	2747	10.6
**Socioeconomic status**						
Low	30,754	28.4	23,192	28.2	7562	29.2
Medium	43,224	39.9	33,257	40.4	9967	38.5
High	34,284	31.7	25,942	31.5	8333	32.2
**Immunohistochemistry subtype**						
HR+/HER2-	68,806	63.6	52,891	64.2	15,915	61.5
HR+/HER2+	7141	6.6	5030	6.1	2115	8.2
HR-/HER2+	3985	3.7	2730	3.3	1255	4.9
HR-/HER2-	8516	7.9	5846	7.1	2670	10.3
Unknown	19,805	18.3	1,5894	19.3	3911	15.1
**Late-stage BC**						
Early BC	94,988	87.8	75,236	91.3	19,752	76.4
Advanced BC	13,265	12.3	7155	8.7	6110	23.6
**Late-stage BC**						
Non-metastatic BC	104,395	96.4	81,004	98.3	23,391	90.5
Metastatic BC	3858	3.6	1387	1.7	2471	9.6

BC, breast cancer. HR, hormone receptor. HER2, human epidermal growth factor receptor 2.

**Table 2:** Characteristics of women and their breast cancer by screen-detected status.

	Total (n = 108,253)		Screen-detected BC (n = 62,164)		Non-screen-detected BC (n = 46,089)	
	n	%	n	%	n	%
**Age at diagnosis**						
49–54	27,794	25.7	13,889	22.2	13,905	30.2
55–59	19,404	17.9	10,758	17.3	8646	18.8
60–64	22,125	20.4	13,132	21.1	8993	19.5
65–69	21,682	20.0	13,514	21.7	8168	17.7
70–74	17,248	15.9	10,871	17.5	6377	13.8
**Year**						
2007	9413	8.7	5107	8.2	4306	9.3
2008	9666	8.9	5255	8.5	4411	9.6
2009	9901	9.1	5331	8.6	4570	9.9
2010	10,293	9.5	5855	9.4	4438	9.6
2011	10,801	10.0	6223	10.0	4578	9.9
2012	11,315	10.5	6677	10.7	4638	10.1
2013	11,677	10.8	7035	11.3	4642	10.1
2014	11,640	10.8	6760	10.9	4880	10.6
2015	11,712	10.8	6957	11.2	4755	10.3
2016	11,835	10.9	6964	11.2	4871	10.6
**Socioeconomic status**						
Low	30,754	28.4	17,639	28.4	13,115	28.5
Medium	43,224	39.9	25,051	40.3	18,173	39.4
High	34,284	31.7	19,474	31.3	14,801	32.1
**Immunohistochemistry subtype**						
HR+/HER2-	68,806	63.6	39,994	64.3	28,812	62.5
HR+/HER2+	7141	6.6	3400	5.5	3741	8.1
HR-/HER2+	3985	3.7	1534	2.5	2451	5.3
HR-/HER2-	8516	7.9	3093	5.0	5423	11.8
Unknown	19,805	18.3	14,143	22.8	5662	12.3
**Late-stage BC**						
Early BC	94,988	87.8	59,121	95.1	35,867	77.8
Advanced BC	13,265	12.3	3043	4.9	10,222	22.2
**Late-stage BC**						
Non-metastatic BC	104,395	96.4	61,758	99.4	42,637	92.5
Metastatic BC	3858	3.6	406	0.7	3452	7.5

BC, breast cancer. HR, hormone receptor. HER2, human epidermal growth factor receptor 2.

### 3.2 Incidence of overall, late-stage, and early stage of BC

**Fig. 2:**
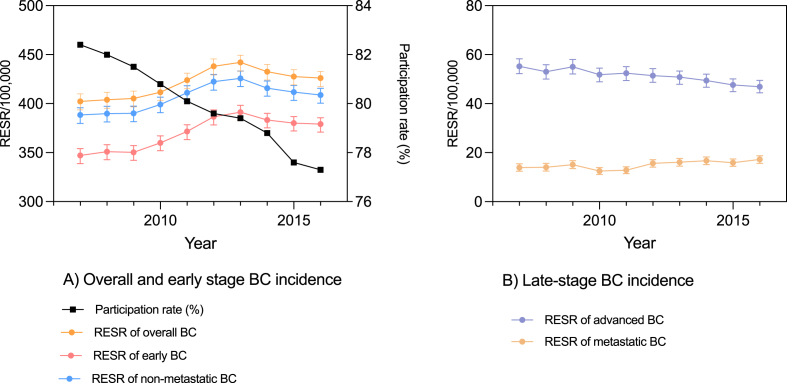
Breast cancer incidence standardised by Revised European Standardized Rate and participation rate during 2007 and 2016 A) Overall and early stage BC incidence; B) Late-stage BC incidence RESR, revised European standardized incidence rates. BC, breast cancer.

The age-standardized incidence rates for overall BC exceeded 400 per 100,000 women from 2007 onward (Fig. 2A). Overall, early BC and non-metastatic BC at diagnosis showed comparable trends, increasing between 2007 and 2013, then decreasing between 2013 and 2016. Between 2007 and 2016 the incidence of late-stage BC decreased from 55.2 to 46.9 per 100,000 women, while metastatic BC persisted below 20 per 100,000 women, increasing slightly from 13.9 to 17.2 (Fig. 2B). Participation in the BCSP decreased from 82.4% in 2007 to 77.3% in 2016 (Fig. 2A).

### 3.3 Screen-related and screen-detected BC diagnosis in relation to late-stage

In multivariable analysis, the odds of advanced BC were 3.24 times higher in the non-screen-related BC group compared with the screen-related BC group (aOR = 3.24, 95% CI = 3.12–3.37) (Table 3). In multivariable analysis, the odds of metastatic BC were 6.40 times higher in the non-screen-related BC group compared with the screen-related BC group (aOR = 6.40, 95% CI = 5.98–6.85).

**Table 3:** Screen-related versus non–screen-related breast cancer and late-stage presentation: univariate and multivariable analysis (OR and 95% CI).

	Univariate OR (95%CI)		Adjusted OR (95%CI)^a^	
	**Advanced BC**	**Metastatic BC**	**Advanced BC**	**Metastatic BC**
**Screening-related BC**				
Yes	1	1	1	1
No	3.25 (3.13-3.38)	6.17 (5.77-6.60)	3.24 (3.12–3.37)	6.40 (5.98–6.85)
**Age**	0.99 (0.99-0.99)	1.00 (1.00-1.01)	1.00 (1.00-1.00)	1.02 (1.01-1.02)
**SES**				
Low	1	1	1	1
Medium	0.93 (0.89-0.97)	0.89 (0.82-0.96)	0.95 (0.91-0.99)	0.93 (0.86-1.00)
High	0.86 (0.82-0.90)	0.81 (0.75-0.88)	0.85 (0.81-0.89)	0.83 (0.76-0.90)

OR, odds ratio. BC, breast cancer. SES, socio-economic status.

a Adjusted ORs were estimated using multivariable analyses.

In multivariable analysis, the odds of advanced BC were 5.54 times higher in the non-screen-detected BC group compared with the screen-detected BC group (aOR = 5.54, 95%CI = 5.31–5.78) (Table 4)*.* In multivariable analysis, the odds of metastatic BC were 12.66 times higher in the non-screen-detected BC group compared with the screen-detected BC group (aOR = 12.66, 95% CI = 11.41–14.05).

**Table 4:** Screen-detected versus non–screen-detected breast cancer and late-stage presentation: univariate and multivariable analysis (OR and 95% CI).

	Univariate OR (95%CI)		Adjusted OR (95%CI)^a^	
	**Advanced BC**	**Metastatic BC**	**Advanced BC**	**Metastatic BC**
**Screen-detected BC**				
Yes	1	1	1	1
No	5.54 (5.31-5.78)	12.32 (11.10-13.66)	5.54 (5.31–5.78)	12.66 (11.41–14.05)
**Age**	0.99 (0.99-0.99)	1.00 (1.00-1.01)	1.00 (1.00-1.00)	1.02 (1.01-1.02)
**SES**				
Low	1	1	1	1
Medium	0.93 (0.89-0.97)	0.89 (0.82-0.96)	0.94 (0.89-0.98)	0.90 (0.84-0.98)
High	0.86 (0.82-0.90)	0.81 (0.75-0.88)	0.84 (0.80-0.88)	0.81 (0.74-0.88)

OR, odds ratio. BC, breast cancer. SES, socio-economic status.

a Adjusted ORs were estimated using multivariable analyses.

### 3.4 Screen-related and screen-detected BC diagnosis in relation to late-stage by subtype

**Fig. 3:**
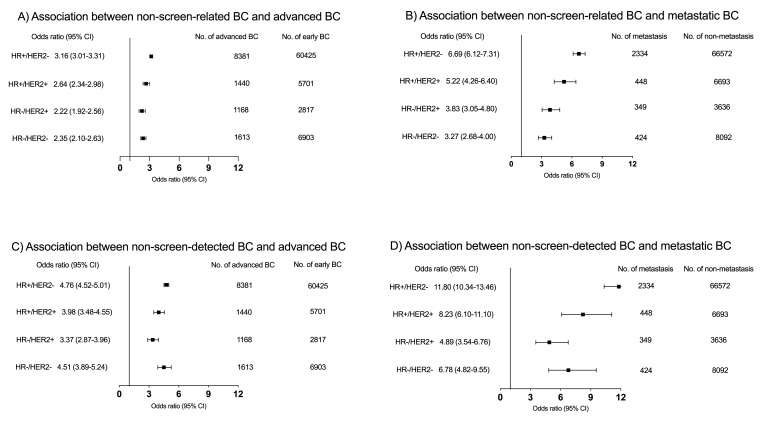
Forest plot of the associate between screen-related/detected BC and late-stage by immunohistochemistry-based subtype A) Association between non-screen-related BC and advanced BC; B) Association between non-screen-related BC and metastatic BC; C) Association between non-screen-detected BC and advanced BC; D) Association between non-screen-detected BC and metastatic BC. BC, breast cancer. HR, hormone receptor. HER2, human epidermal growth factor receptor 2.

The associations of screen-related and screen-detected BC with advanced and metastatic disease were stratified by immunohistochemistry-based subtype (Fig. 3). HR+/HER2− tumours, the most prevalent subtype (n = 68,719), showed elevated ORs of advanced and metastatic disease, with adjusted ORs 3.16 (95% CI = 3.01–3.31) and 6.69 (95% CI = 6.12–7.31) for non-screen-related BC, and adjusted ORs of 4.76 (95% CI = 4.52–5.01) and 11.80 (95% CI = 10.34–13.46) for non-screen-detected BC, respectively. (Supplemental Table 1).

## 4 Discussion

The overall incidence of BC in the Netherlands increased between 2007 and 2013 and then declined each year to 2016. The non-screen-related group had higher odds of being diagnosed with late-stage BC than the screen-related group. Similar results were observed for non-screen-detected BCs. This association was observed across all immunohistochemistry-based subtypes, with the strongest effect seen for HR+/HER2− tumours.

Over the decade from 2007 to 2016 the incidence of advanced BC decreased whereas that of metastatic BC remained stable. This most likely reflects increased early BC detection through screening. Additionally, a shift from film mammography to digital mammography that started in 2003 and completed in 2010, also contributed to this trend. Digital mammography detects smaller tumours and DCIS [31], explaining the earlier initial increase within 2007-2014 and later decline in early BC within 2013-2016. Screening and digital mammography explain lower advanced BC incidence; however, this is unlikely to affect the incidence of metastatic BC, which remained stable. Additionally, since de-novo metastatic BC diagnoses represent a minority of BC cases and most metastatic BC occurs as recurrence, it would be difficult to detect subtle effects in this subgroup. These results mirror trends observed in Sweden [32] and Norway [33]. In Sweden, stage III BC diagnoses were shown to decrease from 15% in the period 1989–1993 to 12% in the period 2009–2013, with a 48% reduction in 5-year excess mortality [32]. In Norway, the incidence of early stage BC also increased from 113.7 per 100,000 in 1996–2004 to 141.2 per 100,000 in 2005–2010 among women aged 50–69 years, while the incidence of stage II + BC decreased slightly [33].

Our study showed that the odds of late-stage BC were higher in the non-screen-detected BC group, consistent with previous studies. Ding et al. reported that risk increased with decreasing participation, with ORs of irregularly screened, single screened, and non-attenders compared to regularly screened women were 1.17, 2.18, and 5.95, respectively [15]. A study in Sweden also reported a 25% lower risk of advanced BC (defined as invasive breast cancer measuring >20 mm and/or with ≥4 metastatic axillary lymph nodes) among attendees compared with non-attendees [7]. In Austria, women exposed to screening experienced a 17% lower incidence of advanced BC and 73% lower incidence of metastatic BC compared with women not exposed to screening [8]. Similarly, attendees in Italy had 39% lower odds of stage T2 or larger tumours compared with non-attenders [34].

Some conflicting evidence also exists regarding the role of screening in reducing the incidence of advanced BC. A study from Lithuania found no significant change from before to after screening implementation [35], and a 17-year study in Denmark also showed no decline in the incidence of advanced BC [14]. However, both studies used aggregated rather than individual level data, potentially explaining the different outcomes compared with our research. Moreover, the study in Lithuania had a low participation rate (18%–45%) that limited assessment of the impact of screening [35]. The study in Denmark did not account for socioeconomic differences between screening and non-screening areas, which could have affected the background incidence of advanced BC due to differences in risk factors and participation rates.

The associations were observed across all HR/HER2-defined subtypes. However, the strongest associations were observed for HR+/HER2-cancers, consistent with their more indolent biological behaviour [36]. These tumours typically have lower proliferation rates and longer tumour doubling times, resulting in a prolonged preclinical detectable phase that favours detection by screening [37,38]. In contrast, more aggressive subtypes, such as HR-/HER2+ and triple-negative breast cancer may progress rapidly [37], reducing stage differences between screen and non-screen-detected or related cases.

Unlike screen-detected BC, which is widely used in the literature [34,39,40], the term screen-related BC is less commonly used. In our study, screen-related BCs were defined based on the timing of diagnosis relative to screening participation, including both screen-detected and interval cancers, consistent with a previous Dutch study [17]. This approach evaluates the overall performance of organized screening programs rather than only positive screening tests. Similar classification systems, such as in Finland [41], distinguish cancers by detection mode and screening participation. Our definition aligns with this framework and enables assessment of the program's impact on late-stage breast cancer incidence.

Few studies have directly linked screening behaviour to a cancer registry but have instead relied on aggregated data on overall screening attendance by region [12,42,43]. We linked the BCSP database with the NCR, allowing classification of BC diagnoses as screen-related, non-screen-related, screen-detected, or non-screen-detected, and included TNM information for all Dutch BC patients. Therefore, our approach provided a more comprehensive and individualized perspective. Even in Nordic countries attendance rate rarely exceeds 90%, indicating importance of accounting for individual-level variability in screening uptake, rather than assuming universal participation based on program implementation alone [14].

The incidence of late-stage BC can be used as a proxy for BC mortality [6], with reductions in BC mortality depending on decreasing the incidence of late-stage BC and improved treatment. Therefore, changes in the incidence of late-stage BC can be used to assess the impact of screening, with effective screening leading to reduced rates of late-stage diagnoses, hence fewer BC-related deaths [6,26]. Although advancements in treatment can improve survival rates, screening remains crucial to extending life expectancy through early detection [44].

This study has several strengths. First, we established a link between screening and cancer diagnosis for each patient providing more precise assessment of screening effects thus avoiding use of aggregated data. Second, few studies have provided nationwide coverage [19,45], whereas we included all eligible Dutch women to enhance generalizability [46]. Third, changes to the TNM staging system during the study were minor and did not affect stage classification. Defining late-stage stage using the TNM system rather than tumour size enhances clinical relevance for treatment and survival assessment. The thresholds for late-stage BC definitions align with clinical practice, supporting treatment decisions compared to previous research.

This study has also limitations. Information on women with high-risk characteristics, such as family history of BC, was not available in our dataset, where about 17.8% are known to carry a (likely) pathogenic variant [47]. Their participation in additional or alternative screening programs may have resulted in misclassification of screening status and, consequently, an overestimation of the effects attributed to non-screening. Second, the available data were limited to the period up to 2016, which was the most recent year for which detailed information on screening type was available at the time of writing. Furthermore, as the Dutch Cancer Registry is tumour-based rather than person-based, we were unable to assess the impact of prevalent screens. Data on place of living was not available, and the role of socio-economic status was not comprehensively examined in this study. Future research should investigate this aspect in greater depth to better understand its potential impact in the Dutch BCSP.

## 5 Conclusion

The incidence of late-stage BC is lower among screen-related cancers compared with non-screen- related cancers, regardless of the definition used for late-stage BC. Moreover, this pattern is also observed among screen-detected cancers compared with non-screen-detected cancers and is consistently across all immunohistochemistry subtypes. Our findings underscore the need for well-implemented BCSPs with high participation rates.

## CRediT authorship contribution statement

**Yixuan Lin:** Writing – review & editing, Writing – original draft, Visualization, Formal analysis, Data curation. **Grigory Sidorenkov:** Writing – review & editing, Supervision, Data curation. **Jing Wang:** Writing – review & editing, Methodology, Conceptualization. **Marcel J.W. Greuter:** Writing – review & editing, Supervision, Data curation. **Nehmat Houssami:** Writing – review & editing. **Monique D. Dorrius:** Writing – review & editing. **Sabine Siesling:** Writing – review & editing, Resources. **Linda de Munck:** Writing – review & editing, Supervision, Resources, Methodology. **Geertruida H. de Bock:** Writing – review & editing, Supervision, Project administration, Methodology, Conceptualization.

## Funding

This work was supported by China Scholarship Council (CSC) through a PhD scholarship awarded to YL (grant No.202107930008). NH receives funding via a National Breast Cancer Foundation (NBCF) Chair in Breast Cancer Prevention (grant No. EC-21-001).

## Declaration of competing interest

NH declares being a member of the BreastScreen Australia National Policy Review, Expert Advisory Group (2023–25).

Given her roles as Editorial Board Member, GHdB and SS had no involvement in the peer-review of this article and have no involvement in the peer review of this article and have no access to information regarding its peer review.

## Data Availability

The datasets analyzed during the current study are not publicly available due to privacy regulations. The dataset can be made available via the NCR (https://iknl.nl/en/ncr/apply-for-data) after request and approval of a proposal.
