# Supplementary material for: Impact of screening on late-stage breast cancer in the Netherlands: a population-based cohort study (2007-2016)
Source: Breast. 2026 Mar 2;86:104745. doi: 10.1016/j.breast.2026.104745 (PMC12972954; doi:10.1016/j.breast.2026.104745)
Supplement: Multimedia component 1 [file mmc1.docx]

Supplemental Table 1. Non-screen-detected and non-screen-related breast cancer and late-stage breast cancer (advanced BC and metastatic BC), stratified by HR/HER2-defined subtypes: **multivariable analysis (adjusted OR and 95% CI) ***

|  | Adjusted OR (95%CI) * | | | |
| --- | --- | --- | --- | --- |
|  | Non-screen-related and advanced BC | Non-screen-related and metastatic BC | Non-screen-detected and advanced BC | Non-screen-detected and metastatic BC |
| Overall  (n=108,253) | 3.24  (3.12–3.37) | 6.40  (5.98–6.85) | 5.54  (5.31–5.78) | 12.66  (11.41–14.05) |
| HR+/HER2- | 3.16  (3.01-3.31) | 6.69  (6.12-7.31) | 4.76  (4.52-5.01) | 11.80  (10.34-13.46) |
| HR+/HER2+ | 2.64  (2.34-2.98) | 5.22  (4.26-6.40) | 3.98  (3.48-4.55) | 8.23  (6.10-11.10) |
| HR-/HER2+ | 2.22  (1.92-2.56) | 3.83  (3.05-4.80) | 3.37  (2.87-3.96) | 4.89  (3.54-6.76) |
| HR-/HER2- | 2.35  (2.10-2.63) | 3.27  (2.68-4.00) | 4.51  (3.89-5.24) | 6.78  (4.82-9.55) |

BC, breast cancer. OR, odds ratio. HR, hormone receptor. HER2, human epidermal growth factor receptor 2.

* Adjusted ORs were estimated using multivariable analyses and were adjusted for age and socioeconomic status.
